# Using the intervention mapping protocol to develop a community-based intervention for the prevention of childhood obesity in a multi-centre European project: the IDEFICS intervention

**DOI:** 10.1186/1479-5868-8-82

**Published:** 2011-08-01

**Authors:** Vera Verbestel, Stefaan De Henauw, Lea Maes, Leen Haerens, Staffan Mårild, Gabriele Eiben, Lauren Lissner, Luis A Moreno, Natalia Lascorz Frauca, Gianvincenzo Barba, Éva Kovács, Kenn Konstabel, Michael Tornaritis, Katharina Gallois, Holger Hassel, Ilse De Bourdeaudhuij

**Affiliations:** 1 Department of Movement and Sport Sciences, Ghent University, Watersportlaan 2, Ghent 9000, Belgium; 2 Department of Public Health, Ghent University, De Pintelaan 185, block A, Ghent 9000, Belgium; 3 Research Foundation Flanders, Ghent University, Watersportlaan 2, 9000 Ghent, Belgium; 4 Department of Pediatrics, The Queen Silivia Childrens' University Hospital, Göteborg University, Smörslottsgatan, 41685 Göteborg, Sweden; 5 Department of Public Health and Community Medicine, Sahlgrenska Academy, Box 454, University of Gothenburg, 40530 Göteborg, Sweden; 6 GENUD (Growth, Exercise, Nutrition and Development) Research Group, University School of Health Sciences, University of Zaragoza, Domingo Miral s/n, Zaragoza 50009, Spain; 7 Institute of Food Sciences, Unit of Epidemiology and Population Genetics, National Research Council, Via Roma 52 AC, Avellino 83100, Italy; 8 Department of Pediatrics, University of Pécs; Jozsef A. str. 7, H-7623 Pécs, Hungary; 9 National Institute for Health Development, Hiiu 42, Tallinn 50410, Estonia; 10 Research and Education Institute for Child Health, Attikis str. 8, Strovolos 2027, Cyprus; 11 Bremen Institute for Prevention Research and Social Medicine (BIPS), University of Bremen, Achterstr. 30, Bremen 28359, Germany; 12 Hochschule Coburg, University of Applied Sciences, Friedrich-Streib-Str. 2, 96450 Coburg, Germany

## Abstract

**Background:**

The prevalence of childhood obesity has increased during the past decades and is now considered an urgent public health problem. Although stabilizing trends in obesity prevalence have been identified in parts of Europe, preventive efforts in children are still needed. Using the socio-ecological approach as the underlying theoretical perspective, the IDEFICS project aimed to develop, implement and evaluate a community-based intervention for the prevention of childhood obesity in eight European countries. The aim of the present manuscript was to describe the content and developmental process of the IDEFICS intervention.

**Methods:**

The intervention mapping protocol (IMP) was used to develop the community-based intervention for the prevention of childhood obesity in 3 to 10 years old children. It is a theory- and evidence-based tool for the structured planning and development of health promotion programs that requires the completion of six different steps. These steps were elaborated by two coordinating centers and discussed with the other participating centers until agreement was reached. Focus group research was performed in all participating centers to provide an informed basis for intervention development.

**Results:**

The application of the IMP resulted in an overall intervention framework with ten intervention modules targeting environmental and personal factors through the family, the school and the community. The summary results of the focus group research were used to inform the development of the overall intervention. The cultural adaptation of the overall intervention was realised by using country specific focus group results. The need for cultural adaptation was considered during the entire process to improve program adoption and implementation. A plan was developed to evaluate program effectiveness and quality of implementation.

**Conclusions:**

The IDEFICS project developed a community-based intervention for the prevention of childhood obesity by using to the intervention mapping heuristic. The IDEFICS intervention consists of a general and standardized intervention framework that allows for cultural adaptation to make the intervention feasible and to enhance deliverability in all participating countries. The present manuscript demonstrates that the development of an intervention is a long process that needs to be done systematically. Time, human resources and finances need to be planned beforehand to make interventions evidence-based and culturally relevant.

## Background

The prevalence of overweight and obesity in Europe has increased during the past decades [1,2] and is considered a significant public health problem [2]. This worrying trend has not only been evident among European adolescents and adults but has also been identified in children below the age of 10 [1-3]. Although the prevalence of childhood obesity is stabilizing in some European countries [4,5], the prevalence is still alarming because childhood obesity is related with adverse health consequences [6] and tends to persist into adulthood [7,8]. As the prevalence of childhood obesity remains generally high, especially in groups with a lower socio-economic status (SES) [5], preventive efforts in children are still needed.

Evidence already indicated that school-based interventions can be effective in the prevention of overweight but to date, the majority of childhood obesity prevention efforts described in the literature have been unsuccessful [9-11]. Furthermore, there is a growing recognition that childhood obesity should be prevented by using a global socio-ecological approach. According to the socio-ecological approach, effective behavioral change can be obtained by targeting the ecological environment of the child which includes the family, the school and the community at large and by targeting psychological, socio-cultural, policy and physical environmental factors [12-15]. However, the use and evaluation of multilevel approaches in the prevention of childhood obesity is rare [9,16]. The IDEFICS (Identification and prevention of Dietary- and lifestyle-induced health EFfects In Children and infantS) project aims to counter the lack of an ecological approach in previous intervention-based research [17]. Therefore a main purpose of the IDEFICS project is to develop, implement and evaluate a community-based preventative intervention program in 2-10 year old children in eight different European countries (Belgium, Cyprus, Estonia, Germany, Hungary, Italy, Spain, Sweden) [18,19].

Because the literature previously called for a structured and evidence-based development of intervention programs [20], the intervention mapping protocol (IMP) was used as the theoretical framework for the development of the IDEFICS intervention. The IMP is a problem- and theory-driven protocol that was especially developed to guide the design of evidence-based intervention programs [13]. It also recognizes the importance of a socio-ecological approach in behavioural change [13,20] which was of particular importance in the present project. Furthermore, the IMP aids and necessitates the detailed description of intervention content which meets recent demands for more thorough reporting on what happens in intervention-based research [21].

The present paper will describe and inform program planners about the process of developing an intervention program in a multi-centre European project by using the intervention mapping heuristic.

## Methods

The IDEFICS intervention has been developed according to the IMP. This protocol describes the process for developing theory- and evidence-based intervention programs [13] and consists of six different steps: 1) needs assessment, 2) formulation of change objectives, 3) selection of theory-based methods and practical strategies, 4) development of the intervention program, 5) development of an adoption and implementation plan, and 6) development of an evaluation design. This paper briefly explains the core processes of the protocol and a more comprehensive overview of the IMP can be found at http://interventionmapping.com.

Two out of the eight intervention centers were responsible for coordinating and developing the IDEFICS intervention (Ghent University and University of Gothenburg). Draft versions of the elaborated intervention mapping steps (excluding step 2 and 3) were discussed with the other intervention centers until agreement was reached. In total, 24 months were available for the development of the intervention. The process of developing an intervention in a multi-centre European project according to the intervention mapping heuristic within this timeframe is outlined in Table 1 and described in more detail below.

**Table 1:** Timeline of intervention development activities during the preparation phase of the project (September 2006 - August 2008)

YEAR 1: SEPTEMBER 2006 - AUGUST 2007											
		
SEP OCT NOV DEC				JAN FEB MAR APR MAY JUN JUL							AUG
		
Literature review by the main coordinating centre Development of focus group protocol by main coordination centre				Conduction of focus groups in all intervention centers Elaboration of the Needs Assessment (step 1) by the coordinating intervention centers							FIRST face-to-face meeting with all intervention centers: - Agreement upon step 1 - Brainstorming about the change objectives (step 2), the selection of theory-based methods and practical strategies (step 3), the program development (step 4) and the adoption and implementation plan (step 5)
**YEAR 2: SEPTEMBER 2007 - AUGUST 2008**											
				
SEP OCT		NOV	DEC	JAN	FEB MAR APR MAY JUN JUL AUG						
				
Elaboration of step 2-5 by the coordinating intervention centers		SECOND face-to-face meeting with all intervention centers: - Agreement upon step 4 and 5 - Checking the conformity of the intervention modules with the focus groups results in all intervention centers Discussing the feasibility of adoption and implementation of the intervention in all intervention centers	Finalization of step 4 and 5 by the coordinating intervention centers	CENTRAL training on intervention activities: - Fine tuning of the intervention between centers - Discussion of opportunities for cultural adaptation Discussion of draft version of process evaluation instruments	Local training(s) in each intervention centre Reporting the plans for cultural and local adaptation in written form to the coordination centers Preparation of local intervention adaptation and implementation Further development and agreement about process evaluation instruments by e-mail and telephone conferences						

### Step 1: Needs assessment

In the first step of the protocol, the health problem is analyzed, followed by a study of the related risk behaviours and its determinants [13]. The needs assessment of the present study was focused on the target group of the IDEFICS project (i.e. 3 to 10 years old children) and included an analysis of the literature on the determinants and correlates of childhood obesity, the role of predefined behavioral risk factors in the development of childhood obesity (i.e. physical activity, dietary behavior and stress) and its related determinants. Further, the literature reporting on effective interventions in the prevention of childhood obesity was analyzed. This literature analysis was done by the main coordinating center.

In addition, focus group interviews were conducted in all countries with children, parents of different socio-economic backgrounds, teachers and community leaders to identify local barriers, difficulties and influencing factors of the predefined target behaviors. The focus group protocol was developed and coordinated across the intervention centers by the main coordinating centre and finalized together with all participating centers. A detailed description of the protocol can be found elsewhere [22,23]. A first face-to-face meeting with personnel from all intervention centers was held in August 2007 to discuss the results of the needs assessment (Table 1) and to agree upon the behavioral program objectives. This face-to-face meeting was also used to brainstorm about the subsequent intervention mapping steps.

### Step 2: Formulation of change objectives

In the second step of the protocol, each program objective was subdivided into performance objectives. These objectives are the expected sub behaviours that have to be accomplished by the target group to achieve the program objective. By crossing the determinants with the performance objectives, the more general performance objectives were translated into very specific intervention objectives, i.e. the change objectives. Change objectives were formulated for each program objective and were formulated by the coordinating centres.

### Step 3: Selection of theory-based methods and practical strategies

The third step of the IMP includes the identification and selection of theoretical methods considered to influence changes in the selected determinants [13]. During this selection process, the summary of theoretical methods provided by Bartholomew and colleagues [13] was used. In a next step, practical strategies had to be identified to put the theoretical methods into practice [13]. Special efforts were made to search for and select existing strategies that fitted with the theoretical methods and specific intervention objectives. The summary results of the focus groups were used to inform the selection of existing strategies and the development of new strategies. This intervention mapping step was elaborated by the coordinating centers.

### Step 4: Program development

In this step of the IMP, the information from all previous steps was combined with the intervention program as the final result [13]. A proposal for the content of the IDEFICS intervention was made by the coordinating centers. This was discussed with all IDEFICS partners during a second face-to-face meeting in November 2007 (Table 1). During this meeting, attention was paid to the fact that the overall intervention and/or specific intervention components were in line with the focus group results in all centers. Additionally, the feasibility of adopting and implementing the program in all centers was discussed.

### Step 5: Adoption and implementation

The focus of the fifth step of the protocol is the planning of program adoption and implementation, including the consideration of program sustainability [13]. This step of the protocol was supported and informed by the focus group results indicating that the IDEFICS intervention had to be flexible enough to deal with the variability in local circumstances between and within countries [22,23]. Agreement about the strategy for program adoption and implementation was reached during the second face-to-face meeting in November 2007 and finilised by the beginning of 2008 (Table 1). In January 2008, a central training was organised in one of the coordinating centers to finetune all intervention components between centres and to discuss opportunities for cultural and local adaptation. In the months after the central training, all intervention centres planned the adoption of the intervention by the local stakeholders. Plans for cultural and local adaptations made during these preparatory months, were reported in written form to the main coordination centre. In the months before the start of the intervention, all centres organised local training(s) for the research staff being responsible for the adoption of the IDEFICS intervention in the local community.

### Step 6: Evaluation design

In the last step of the IMP, program planners develop a plan to evaluate the effectiveness and to assess the quality of intervention implementation [13]. In contrast to the sequence of intervention mapping steps, the evaluation design was already defined by the start of the European project.

The process evaluation was developed by the main coordinating center as soon as agreement about the intervention content was reached (November 2007). The development of the process evaluation instruments was based on the model of Saunders et al. [24]. During the central training in January 2008, draft versions of the process evaluation instruments were discussed with all intervention centers. Final agreement about the process evaluation instruments was reached through e-mail communication and telephone conferences (Table 1).

## Results

### Step 1: Needs assessment

The literature search revealed that socio-economic status (SES) is an important correlate of body weight [25,26]. Several studies found that children from a lower socio-economic background are at higher risk for the development of obesity [25,27]. Consequently, SES needs to be considered as an important factor in the prevention of childhood obesity. It was also concluded from the literature that specific physical activity, dietary and stress related behaviors are associated with the development of childhood obesity.

The needs assessment resulted in a selection of two key behaviors for each predefined behavior. These key behaviors were translated into six *program objectives *(Table 2): (1) increasing daily physical activity levels, (2) decreasing daily television (TV) viewing time, (3) increasing the consumption of fruit and vegetables, (4) increasing the consumption of water, (5) strengthening parent-child relationships and (6) establishing adequate sleep duration patterns. All program objectives (except for the second) were positively phrased to avoid negative associations to those objectives and to the overall IDEFICS intervention. The rationale for the selection of these program objectives is described below.

**Table 2:** Specific program objectives of the IDEFICS intervention

Physical activity	1. Increasing daily physical activity levels
	2. Decreasing daily TV viewing time
Diet	3. Increasing daily consumption of fruit and vegetables
	4. Increasing daily consumption of water

Stress	5. Strengthening parent-child relationships
	6. Establishing adequate sleep duration patterns

#### Increasing daily physical activity levels and decreasing TV viewing time

Physical activity and sedentary behavior are two components of energy expenditure that contribute to the development of childhood obesity [28,29]. Several studies demonstrated that higher levels of physical activity during early childhood are protective in developing body fat [30-34]. A recent literature review from Monasta and colleagues [35] reported that less than 30 minutes of daily physical activity at preschool age is associated with an increased risk for overweight and obesity

TV viewing is a sedentary behavior consistently being associated with the development of childhood obesity. The reduction of this behavior is suggested to be one of the more successful ways to prevent childhood obesity [28,36-38]. For example, Reilly et al. [38] found that watching more than eight hours TV per week at the age of three is independently related with the risk of obesity. The association between watching TV and childhood obesity is possibly mediated by an increased energy intake in children [28,36,37], underlining the need to target TV viewing as a sedentary risk behavior in the prevention of childhood obesity [37].

#### Increasing daily consumption of fruit, vegetables and water

As large portions of energy-dense foods are found to be positively associated with obesity in early childhood [39], promoting low-energy dense foods might be a promising approach for the prevention of childhood obesity [40,41]. The review of Libuda and Kersting [42] summarized the available evidence on the positive association between childhood obesity development and sugar-sweetened beverage consumption. Because of this association and the recommendation of the IMP to target health-promoting behaviors (i.e. the opposite of the risk behavior) [13], it was decided to replace sugar-sweetened beverages by a non-caloric alternative and to select water consumption as one of the dietary behaviors to be targeted by the IDEFICS intervention. This decision is supported by a recently conducted randomized controlled cluster trial demonstrating that the promotion of water consumption effectively prevents overweight in elementary school children [43]. At the time of conducting the needs assessment, no convincing evidence of other dietary risk factors of childhood obesity was available [39], however, the consumption of fruit and vegetables was selected as a second dietary related target behavior. This decision was based on the health-promoting behavioral approach endorsed by the IMP and the finding that low-energy dense foods, such as fruit and vegetables, moderate energy intake in young children [40,41,44].

#### Strengthening parent-child relationships and establishing adequate sleep duration patterns

There is currently a growing interest in the role of stress in the development of obesity [45,46]. So far, Koch and colleagues [47] found that children who are exposed to psychological stress in the family are more likely to be obese. Generally, the role of the family in childhood obesity is a growing field of interest [48,49] and considered to be important for children's health [47]. The focus group research indicated that interaction and quality time with parents (playing, helping, stay home with the children and doing things together) is believed to reduce stress in children (unpublished IDEFICS data). Based on face-to-face discussions with the intervention centres, it was therefore decided to address stress in children by strengthening parent-child relationships as a fourth program objective.

Growing evidence also suggests that sleep duration is an important risk factor for the development of childhood obesity [35,38,50-53]. Several studies demonstrated that short sleep duration during childhood, i.e. less than 10 hours a day, is an independent risk factor for childhood obesity [38,50,52].

### Step 2: Formulation of change objectives

The six *program objectives *(Table 2) were subdivided into *performance objectives*. As an illustration, the performance objectives of the first program objective "Increasing daily physical activity levels" are presented in Table 3. These performance objectives were formulated based on the guidelines from the National Association for Sport and Physical Education which is currently the most widely used recommendation for physical activity in young children [54]. By crossing the performance objectives with the selected determinants, change objectives were formulated. As an example, the change objectives for the program objective about daily physical activity levels in relation to parental support and physical activity related practices are presented in Table 3.

**Table 3:** Change objectives (i.e. specific intervention objectives) with the aim to increase children's daily activity levels

Performance objectives	Determinants	
	
	Parental support	Physical activity related policies
Children engage in structured physical activity for at least 60 minutes a day	Parents model physical activity in a structured way Parents provide opportunities for participating in structured physical activities	The community and school setting provide opportunities to be physically active in a structured way The community and school setting organise physical activities in a structured way

Children engage in unstructured physical activity for at least 60 minutes and up to several hour a day	Parents model physical activity in an unstructured way Parents provide opportunities for being physically active in an unstructured way	The community and school setting provide opportunities to be physically active in an unstructured way The community and school setting organise physical activities in a unstructured way

Children are not sedentary for more than 60 minutes at a time except when sleeping	Parents reduce the child's exposure to triggers of sedentary behaviour Parents set rules regarding time spent in sedentary activities	The community and school setting provide alternatives for sedentary behaviours

Children develop competence in movement skills	Parents provide opportunities to develop competence in movement skills	The community and school setting provide opportunities for movement experiences to build on children's movement skills

Children become familiar with different kinds of physical activities	Parents provide opportunities for trying different kinds of physical activities	The community and school setting provide opportunities to try out different kinds of physical activities

### Step 3: Selection of theory-based methods and practical strategies

Table 4 presents the methods that were selected for the development of the intervention. This table also describes how the theoretical methods were translated into practical strategies and how these relate to the levels of the intervention. Furthermore, Table 5 shows how the focus group results informed the selection and design of practical strategies.

**Table 4:** Overview of the selected theoretical methods and practical strategies used in the IDEFICS intervention

Level of the intervention	Methods	Related strategies
*Community level*	Forming coalitions	Development of an organisational structure at the community level stimulate collaboration across different agenda's; technical assistance on action and strategic planning (module 1)
	Policy and media advocacy	Placing the topic on the political agenda; sharing resources; increasing public awareness (module 2)
	Facilitation	Changes in the environment (module 3)

*School level*	Forming coalitions	Development of an organisational structure at the school level; stimulate collaboration across different agenda's; technical assistance on action and strategic planning (module 4)
	Facilitation	Changes in the environment (module 6, 7, 8 and 9)

*Class level*	Alternation of perception (altering the perceptions of pros and cons of the desired behaviour so that children give preference to the desired behaviour) Reinforcement (providing reinforces (e.g. incentives) for the performance of the desired behaviour) Implementation intentions (defining specific plans of action, which specify exactly when (time), where (place) and how (response) to behave in future situations) Goal setting (setting reasonable and challenging goals, goals that are difficult but available within the individual's skill level) Modelling with guided enactment (behavioural change by observing and doing, supported by feedback and rewards)	Classroom and homework related activities (module 5) For example: - practical classroom activities (e.g. tasting games, active movement breaks) - theoretical classroom activities (e.g. teaching children how to set goals) - diaries (registering of the progress of a specific behaviour and reinforcement of the desired behaviour) - creating and evaluating an accomplishment plan for the desired behaviour (children taking home their behavioural goals set during the theoretical lesson and trying to realise their goals with their parents)

		
		
*Family level*	Alternation of perception Modelling with guided enactment Persuasive communication	Homework related activities (module 5) Homework related activities (module 5) Homework related activities (module 5) Educational folders and posters (module 10)

**Table 5:** Association between the focus groups results, the final content of the IDEFICS intervention and the intervention mapping steps

Focus group result(s)	Objective/strategy	Content IDEFICS intervention	Intervention mapping step(s)
Children receive inconsistent messages from family and school (regarding rules and availability of food)	Creating and enhancing uniformity of messages to parents and children by:		Step 1 (Needs assessment) Step 3 (Selection of theory-based methods and practical strategies) Step 4 (Program development)
	- Involving parents in environmental and policy changes at the school level	Module 4: Establishment of the school working groups	
	- Creating a school environment in which healthy eating behaviours are the easiest choice	Module 8: Environmental and policy changes related to water consumption Module 9: Environmental and policy changes related to fruit and vegetable consumption	
	- Involving the schools in the community platform to trigger collaboration between schools in the same community	Module 1: Establishment of the community platform	
	- Learning parents how to create a home environment in which healthy eating behaviours are the easiest choice	Module 10: Educational materials for parents providing strategies to create health promoting family environments	

Interaction and quality time with parents (playing, helping, stay home with the children, doing things together ...) is believed to reduce stress in children	Creating a program objective for the predefined behaviour "stress and relaxation"	The predefined behaviour was translated into "Strengthening parent-child relationships"	Step 1 (Needs assessment)

Differences in overall focus group results were larger within countries than between countries.	Creating a structure that enables adaptation of an overall intervention framework within countries and between countries	Module 1: Establishment of the community platform Module 4: Establishment of the school working groups	Step 5 (Adoption and implementation)

School related policies as a barrier for healthy eating at school (mentioned by the parents)	Creating a school environment in which healthy eating behaviours are the easiest choice	Module 8: Environmental and policy changes related to water consumption Module 9: Environmental and policy changes related to fruit and vegetable consumption	Step 1 - 3
	Involving parents in environmental and policy changes at the school level, communication about food policy to the parents.	Module 4: Establishment of the school working groups	

Only the Belgian and Spanish children mentioned receiving lessons about healthy eating.	Providing ready to use nutrition education lessons that can easily be incorporated into the classroom curriculum, stimulate teachers to daily promote healthy eating.	Module 5: Integration of the key behaviours in the classroom activities and providing related homework activities (curriculum-based)	Step 1 - 3

Parents perceive the schools as an important setting for the promotion of healthy eating and physical activity. Parents assigned the main responsibilities for healthy eating and physical activity promotion outside the family context.	Raising awareness among parents about their own role in promoting healthy eating and facilitate their in their ability to create health promoting family environments	Module 10: Educational materials for parents providing strategies to create health promoting family environments	Step 1 - 3
	Creating a school environment in which healthy eating behaviours are the easiest choice	Module 8: Environmental and policy changes related to water consumption Module 9: Environmental and policy changes related to fruit and vegetable consumption	
	Creating an activity promoting school environment	Module 6: Environmental changes related to physical activity: the active playground Module 7: Health related physical education curricula	

Importance of taste for children's food preferences.	Integrating tasting activities in the classroom activities	Module 5: Integration of the key behaviours in the classroom activities and providing related homework activities (curriculum-based)	Step 1 - 3

Peers are perceived to influence the preferences for certain food items.	Stimulating the eating of healthy products in group, stimulate teachers to be a role model	Module 5: Integration of the key behaviours in the classroom activities and providing related homework activities (curriculum-based)	Step 1 - 3

Media, free booklets and magazines, pamphlets and the food pyramid were channels through which parents receive information regarding healthy eating/living. Controversial opinions were assessed regarding the role of media and television (these channels are perceived to distribute contradictory and less reliable information)	Using the channels mentioned during the focus groups. Making a distinction between the intervention campaign and less reliable or contradictory information provided by certain media by using the IDEFICS logo on all documents.	Module 2:Long term multimedia and public relations campaign	Step 1 - 3

Time spent outside is perceived to be dependent of opportunities to be physically active and neighbourhood safety (e.g. traffic, teenage gangs).	Stimulating community members to negotiate for larger scale actions that increase and improve the opportunities to be physically active (e.g. increasing the number of playgrounds and parks, providing age appropriate recreation areas) and negotiate for the improvement of neighbourhood safety.	Module 1: Establishment of the community platform Module 3: Short and a long term perspective for the prevention of childhood obesity developed by local community members	Step 1 - 3

Parents mentioned a lack of structured physical activities offered for preschoolers.	Stimulating schools to include structured physical activities in preschoolers' weekly/daily program.	Module 7: Health related physical education curricula	Step 1 - 3
	Informing parents about the existing facilities and opportunities and stimulating them to provide these opportunities to their children (e.g. sports club)	Module 10: Educational materials for parents providing strategies to create health promoting family environments	
	Stimulating community member to negotiate for an adequate offer of structured physical activities for preschoolers in the community	Module 3: Short and a long term perspective for the prevention of childhood obesity developed by local community members	

Parents with low socio-economic status (SES) mentioned that organized activities are often too expensive.	Stimulating community leaders to negotiate for opportunities to participate in low-cost activities and the possibilities for reductions or lower prices for low SES families with children.	Module 3: Short and a long term perspective for the prevention of childhood obesity developed by local community members	Step 1 - 3

Parents had the perception that specialized physical education teachers are better role models and more often recognize the health promoting role of physical education than regular classroom teachers.	Providing physical education teachers with physical activity promoting didactic guidelines to increase physical activity during physical education.	Module 7: Health related physical education curricula	Step 1 - 3

Social support by parents or friends was mentioned as a factor that influences the time playing outside.	Informing parents about the importance of their role in stimulating their child to be physically active.	Module 10: Educational materials for parents providing strategies to create health promoting family environments	Step 1 - 3

### Step 4: Program development

Step 4 of the protocol resulted in a final intervention framework considered for implementation in eight participating centers. Behavioral change at the individual level was targeted through the development of intervention modules at the level of the community, the schools (including kindergartens and primary schools) and the family. An overview of the intervention at these levels and the related modules and their respective timing can be found in Table 6. A full description of the IDEFICS intervention modules and centrally provided intervention materials will be made available on the IDEFICS website (http://www.idefics.eu). Information on how the summary results of the focus groups informed the development of the intervention program is presented in Table 5.

**Table 6:** Overview and timing of the IDEFICS intervention modules

	COMMUNITY			SCHOOL						FAMILY
			
	Module 1	Module 2	Module 3	Module 4	Module 5	Module 6	Module 7	Module 8	Module 9	Module 10
***YEAR 2 OF THE PROJECT (LAST 7 MONTHS OF THE PREPARATION PHASE; 2008)***										

FEB	Establishment CP	Preparation by CP								
			
MAR	Establishment CP	Preparation by CP								
			
APR	Establishment CP	Preparation by CP		Establishment SWG	. . . . . . . . . . . . . . . . . Preparation by SWG. . . . . . . . . . . . . . . . .					
			
MAY	Establishment CP	Preparation by CP		Establishment SWG	. . . . . . . . . . . . . . . . . Preparation by SWG. . . . . . . . . . . . . . . . .					
			
JUN		Preparation by CP		Establishment SWG	. . . . . . . . . . . . . . . . . Preparation by SWG. . . . . . . . . . . . . . . . .					
			
JUL		Preparation by CP		Establishment SWG	. . . . . . . . . . . . . . . . . Preparation by SWG. . . . . . . . . . . . . . . . .					
			
AUG		Preparation by CP		Establishment SWG	. . . . . . . . . . . . . . . . . Preparation by SWG. . . . . . . . . . . . . . . . .					

***YEAR 3 OF THE PROJECT (INTERVENTION ADOPTION PHASE; 2008 - 2009) - IMPLEMENTATION OF THE MODULES BY:***										

SEP		CP	CP			SWG	(PE) teachers	SWG	SWG	
			
OCT		CP	CP		Teachers (PA)	SWG	(PE) teachers	SWG	SWG	CP and/or SWG (PA)
			
NOV		CP	CP		Teachers (FG)	SWG	(PE) teachers	SWG	SWG	CP and/or SWG (FG)
			
DEC		CP	CP		Teachers (TV)	SWG	(PE) teachers	SWG	SWG	CP and/or SWG (TV)
			
JAN		CP	CP		Teachers (W)	SWG	(PE) teachers	SWG	SWG	CP and/or SWG (W)
			
FEB		CP	CP		Teachers (PA)	SWG	(PE) teachers	SWG	SWG	CP and/or SWG (PA)
			
MAR		CP	CP		Teachers (FG)	SWG	(PE) teachers	SWG	SWG	CP and/or SWG (FG)
			
APR		CP	CP		Teachers (TV)	SWG	(PE) teachers	SWG	SWG	CP and/or SWG (TV)
			
MAY		CP	CP		Teachers (W)	SWG	(PE) teachers	SWG	SWG	CP and/or SWG (W)
			
JUN		CP	CP		Teachers (SP)	SWG	(PE) teachers	SWG	SWG	CP and/or SWG (SP)
			
JUL		CP	CP							
			
AUG		CP	CP							

Modules: 1) Establishment of the community platform; 2) Long term multimedia and public relations campaign; 3) Short and a long term perspective for the prevention of childhood obesity developed by local community members; 4) Establishment of the school working groups; 5) Integration of the key behaviours in the classroom activities and providing related homework activities; 6) Environmental changes related to physical activity: the active playground; 7) Health related physical education curricula; 8) Environmental and policy changes related to water consumption; 9) Environmental and policy changes related to fruit and vegetable consumption; 10) Educational materials for parents providing strategies to create health promoting family environments

Implementers: CP = community platform; SWG = school working group; (PE) teachers = (physical education) teachers

Topics "Healthy Weeks": PA = physical activity; FG = fruit and vegetable consumption; TV = television viewing; W = water consumption; SP = sleep duration

The intervention at the *community level *consisted of three intervention modules (module 1 to 3). Module 1 aimed at the establishment of a "community platform" which can be considered as a working group in which all local and relevant community members (local municipality, social services and welfare sector, private actors) had to be represented. Special emphasis was placed on the inclusion of community members having access to low SES and/or migration groups. The community platform was responsible for the implementation of all other modules at the community level (module 2 and 3). Module 2 consisted of the execution of a long term multimedia and public relations campaign to make the community aware of the intervention and the key behaviours targeted by the intervention. Module 3 involved the development of a short and a long term perspective for the prevention of childhood obesity to establish and induce environmental and policy interventions in the community. The short term perspective required that the community platform developed and implemented a list of obesity preventive actions within the timeframe of the IDEFICS adoption period, i.e. the first year of the intervention (year 3 of the project from September 2008 till August 2009). The long term perspective of the IDEFICS intervention required the development of a list of obesity preventive actions that were not feasible to be accomplished during the adoption period and/or the stated time-limits of the project, mostly for reasons that relate to the time that is realistically required for integrating such actions in the policy implementation plans of communities. However, the community platform was asked to start advocating for the actions defined as part of the long term perspective. Table 7 presents a non-comprehensive list of possible obesity preventive actions that could be taken by the stakeholders of the community platform as part of the short and long term perspective.

**Table 7:** Examples of possible actions that could be undertaken by the stakeholders of the community platform (module 3)

MODULE 3: SHORT AND A LONG TERM PERSPECTIVE FOR THE PREVENTION OF CHILDHOOD OBESITY DEVELOPED BY LOCAL COMMUNITY	
**Possible stakeholders of the community platform**	**Examples of possible actions**

*Local municipality (public health authorities) and local politicians*	- Contribute to national obesity prevention plans - Ensure that all young people have access to youth sports and recreation programs - Promote alternatives for play such as involvement in local organizations (structured activities for children in safe environment for minimal cost) - Support and encourage the development of safe routes in the municipality (especially the routes to schools): include sidewalks/footpaths on all new roads and upgrade the existing roads - Taking vans with physical activity equipment into neighbourhoods that do not have access to physical activity facilities.

*Private sector (food companies, grocery stores)*	- Organisation of shopping tours, grocery taste tests, cooking demonstrations, nutrition labelling - Promote water and healthy food products like fruit and vegetables - Provide easy recipes with fruit and/or vegetables that are typical for a certain season, provide ideas to drink water in several ways (e.g. with a leaflet of mint, with pieces of apple, ...), provide and promote healthy food, e.g. quality fruits and vegetables - Provide healthy options on children's menus

*Working groups of the schools/kindergartens*	- Organise extracurricular physical activity programs - Promote physical activity by disseminating information about community-based sports and recreation programs and help these programs to gain access to school facilities outside of school hours - Enable more after-school care programs to provide regular opportunities for active, physical play - Remove vending machines, particularly soft-drink machines - School pricing incentives that favour low- over high-energy density foods - Promote active commuting to schools (e.g. mapping of safe routes to school, walk/bicycle to school days, walking school buses, bicycle trains)

*Sport and youth organizations*	- Provide and promote free water during the activities - Stimulate the children not to bring sugar sweetened beverages - Stimulate the children to bring fruit and/or vegetables instead of unhealthy snacks - Organise activities in which the family of the children can participate (family events)

*Health care providers*	- Provide assessment, counselling and referral on physical activity, diet, stress, coping and relaxation as part of health care - Encourage parents to be role models for their children in the field of physical activity, diet, stress, coping and relaxation

The intervention at the *school level *consisted of 6 intervention modules (module 4 to 9). Module 4 aimed to establish a school working group in all local participating schools. The school working groups were considered to represent the school and parents' perspective on the intervention program and to provide insight in the realities of working with schools. Therefore, the working groups had to include at least one or more representatives of the school board, several teachers and one or more parent representatives. The school working groups were responsible for the implementation of all other intervention modules at the school level (module 5 to 9). Module 5 consisted of a curriculum-based intervention integrating the key behaviours in the classroom activities. To do so, every participating teacher had to organise eight "Healthy Weeks" during the school year. The timing and initially planned sequence of the Healthy Weeks is shown in Table 6. In each healthy week a specific key behaviour related to nutrition or physical activity was handled and homework was provided to increase involvement of parents. Module 6 focused on environmental changes related to physical activity. For this module, school working groups were invited to create an active playground by providing attractive play tools (e.g. balls, ropes, small bikes) and/or by changing the physical design of the playground (e.g. hopscotch, soccer goal posts, basketball hoops). Module 7 aimed at reaching high(er) activity levels during physical education classes and increasing physical activity levels during the time that children spent in the classroom by providing physical education teachers with practical guidelines. Module 8 and 9 focused on environmental and policy changes related to water and fruit and vegetable consumption respectively. For these intervention modules, school working groups were requested to create a supportive school environment by inducing changes in the school environment and policy (e.g. providing the opportunity to drink water in class, making fruit and vegetables available and accessible in the class room or the school canteen).

The intervention at the *family level *(module 10) consisted of educational materials (posters and flyers) for parents providing them with strategies to remove barriers and facilitate them in their ability to create health promoting family environments.

### Step 5: Adoption and implementation

As the IDEFICS intervention had to be able to deal with the variability in local circumstances between and within countries [22,23], an overall intervention framework with ten different modules was developed, including opportunities for cultural adaptation. The primary aim of integrating opportunities for cultural adaptation was to implement a culturally equivalent version of the overall intervention framework in all participating countries [55]. Opportunities for cultural adaptation were included in the overall intervention framework by the concept of the community platform (module 1) and the school working groups (module 4). These were considered to adapt the overall intervention program to the local and cultural needs within the community and the schools. Examples of how the intervention was culturally adapted in different countries are shown in Table 8.

**Table 8:** Examples of cultural adaptations made to the overall intervention framework

Intervention modules	Examples of cultural adaptations
Module 1: Establishment of the community platform	- Use of existing community platforms instead of creating a new one (e.g. Sweden): the Public Health council and the Child- and Youth steering council were platforms already meeting five to six times a year. These platforms cooperated to act as the community platform of the IDEFICS intervention. - No establishment of a community platform (e.g. Italy): no community platform was created because of the involvement of four different municipalities in the intervention region. To overcome this problem, the school working groups (one for each municipality) were extended with one (or more) representative(s) of the municipality administration (acting on behalf of the Major of the town) and with a representative of the National Health Service (a community pediatrician). One school working group was established in each community because the municipalities involved in the IDEFICS intervention were small towns with one or two primary (plus kindergarten) schools that therefore include the whole population living in the area.

Module 4: Establishment of the school working groups	- Creation of school working groups at the level of the school boards (e.g. Belgium): several schools can be authorized under the same school board. These schools are mostly located at different places in the community. All schools of the same board, have to follow the same school policy which means that they are not independently operating. Therefore, it was not possible to create a school working group in each school and school working groups were created at the level of the school boards. This means that in the Belgian intervention region, 11 school working groups were created representing 21 schools in total. - No establishment of school working groups (Cyprus): as the school system is strictly regulated by the Ministry of education, no changes can be made in schools without going through the Ministry. Therefore, the intervention at school level was not independently implemented from the intervention at the community level. No school working groups were created but they were integrated in the community platform. The platform consists of two main parts: a first part includes the local authorities and stakeholders and a second part includes the authorities of each school. The first part of the platform is taking the decisions while the second part is responsible for the implementation.

Module 5: Integration of the key behaviours in the classroom activities and providing related homework activities	- Adaptations to the timing of the healthy weeks because of a different timing of (summer) holidays across European countries (e.g. start of the healthy weeks in September in Sweden and in October in Belgium). - Adaptation to the sequence of the healthy weeks based on local situations (e.g. Sweden): the healthy week about sleep duration was removed from June to September because an average day in June has about 20 hours of daylight which makes it complicate to talk about sleep duration.

During the first year of the IDEFICS intervention, the intervention was coordinated and supported by the IDEFICS project itself. Therefore, a member of the research staff was appointed as the local "intervention program manager" (IPM) in each participating country. The IPM was responsible for establishing, organizing and coordinating the community platform (module 1) and the school working groups in all participating schools (module 4). The IPM could be a staff member that was involved in the developmental process at the central level, another staff member (not involved at the central level) or a representative person in the local community. If the IPM was not involved in the developmental process at the central level, he/she was informed during local trainings organized within each intervention centre (Table 1).

The community platform was responsible for the local development and implementation of the intervention modules at community (module 2 and 3) and family level (module 10). The school working groups were responsible for the development and implementation of the intervention program at the school level (module 5 to 9). The establishment of the community platform and school working groups varied between countries. In some countries, a community platform and/or school working groups were already in place and therefore could be used and further elaborated according to the IDEFICS format, while other countries had to compile a new platform and/or create new school working groups. The process of establishing new school working groups within the foreseen time-frame (Table 6) was perceived more feasible by the countries than establishing a community platform. The ability of the school working groups to act as implementers of the intervention seemed to be realistic as school working groups seemed to be a commonly used strategy in schools across Europe. The capacity of the platform to act as implementers of the modules at community level varied across countries and was more successful in countries where an existing platform was already available.

The establishment of the community platform and the school working groups enabled adaptation to the local culture and circumstances and ensured the adoption and implementation of the overall intervention framework at all levels of the intervention. However, it has to be noted that the cultural adaptation of the IDEFICS intervention was only allowed at the level of the strategies. For example, the community platform (module 1) was expected to elaborate local initiatives contributing to the prevention of childhood obesity (module 3). Possible actions were centrally provided (Table 7) but the local community platform was allowed to search for and to elaborate other initiatives, as long as the initiatives still fitted with the objectives of the respective module. No central approval was required for the initiatives proposed by the local community platforms. However, the IPM was expected to coordinate and scientifically supervise the community platform. This means that the quality of the intervention was regulated at the level of the countries.

### Step 6: Evaluation design

A quasi-experimental study design was developed to evaluate the effectiveness of the IDEFICS intervention. In each of the eight intervention centers, the intervention region was matched with a comparable control region. Data of about 1000 children (aged 3-10 years old) were collected in each region, comprising data of 2000 children in each country. Data were simultaneously collected in all centers at baseline (T0), 24 months later (T1) and at follow-up (T2). The IDEFICS intervention started in September 2008 and ended in August 2010. The intervention was divided into three stages of program use, referring to the classical phasing of establishing interventions: adoption phase, implementation phase and dissemination phase [13]. The adoption phase was characterized by continuous support and scientific supervision from IDEFICS staff in each country. This input gradually decreased throughout the stages of program use. The effectiveness of the IDEFICS intervention will be evaluated with regard to body mass index, anthropometric measures (primary outcomes) and the behaviors related to the specific program objectives (secondary outcomes). Details about general design, participants, field measurements and related protocols have already been described elsewhere [18,19,56]. The effectiveness of the intervention will also be evaluated with respect to the stages of program use. More specifically, the effectiveness of the adoption phase will be executed by analyzing changes in the outcomes between T0 and T1 in the intervention and control communities and the effectiveness of the implementation phase by analyzing changes in outcomes between T1 and T2. Process evaluation questionnaires were developed for the community members of the community platform, school working groups, teachers and parents. The collection of process evaluation data was synchronized with the survey activities in both control and intervention regions.

## Discussion

The present paper describes the developmental process, content and evaluation design of the IDEFICS intervention. This paper is unique as this process occurred in a multi-centre European context including eight different countries. Furthermore, the IDEFICS intervention focused on six different behaviors through the involvement of the community, the schools and the family which enriches the current literature on intervention-based research aiming at the prevention of childhood obesity. The IMP was used as the conceptual framework for developing the IDEFICS intervention. Due to the foreseen timeframe and the multi-level and multi-behavioral approach of the European project, the protocol was not strictly followed and was reduced in its complexity. This means that the matrices of change objectives were only created at the individual level instead of creating matrices for each level of intervention planning (individual, school, family and the community). However, the roles identified at each ecological level were not neglected but were integrated in the formulation of the change objectives. Despite this reduction in complexity, we believe that the IDEFICS intervention still matches the systematic and evidence-based approach. Though, the development of the intervention was perceived as a time-consuming process. The use of the IMP requires scientific staff, budget and time, and this should be taken into consideration when applying for funds for intervention development. Even if less complex interventions (e.g. fewer behaviors and/or levels) need to be developed, time, financial and human resources should be planned to allow for a systematic and evidence-based development of an intervention.

The external validity of the IDEFICS intervention was an important issue throughout the entire developmental process. As experienced during face-to-face meetings in the present project, this increased the tendency of taking into account very specific and country dependent information during the development of the overall intervention framework. To avoid this tendency, we consider a central coordination as fundamental but acknowledge the importance of continuous contact and consultation with all cooperating centers to guarantee the opportunities and feasibility of local and cultural adaptation.

The focus groups results were used to inform the development of the intervention. However, it was a challenge to find a balance between general information, relevant across the different countries, and very specific, mostly country dependent information. In the present study, the summary results of the focus groups were used to give direction to the content of the intervention while very specific focus group results were applied to culturally adapt the intervention in every participating country. Program planners are advised to emphasize the use of specific focus groups results while preparing for cultural and local adaptation.

Although the literature acknowledges the necessity of multi-component community-based efforts to prevent childhood obesity, we recognize that the school setting still plays a very important role in the IDEFICS intervention as several intervention modules are situated at this level. It is known that schools can have intensive contact with children at a very young age and can reach children with a low(er) SES [57]. Furthermore, the combination of classroom education (module 5), physical activity (module 6) and physical education programs (module 7), changes in the school environment and policy (module 8 and 9) make schools a viable setting for providing obesity interventions in a cost-effective manner [16]. As the literature describes and as confirmed by our formative research, targeting only the school is not the ultimate solution but the role of the school in preventing childhood obesity must be conceptualized as an important part of the broader community intervention [9,58].

Our formative research also demonstrated that, for both physical activity and dietary behaviors, the variability in overall findings was larger within than between countries. This finding provided the opportunity to develop an overall standardized intervention framework for eight participating countries but revealed the need for flexibility to adapt the intervention to the local needs. It should be stressed that the IDEFICS intervention dealt with the variability in local circumstances between and within countries by integrating the establishment of working groups at the community and the school level. This bottom-up approach increases the likelihood of program sustainability in the long term [55]. Furthermore, the IDEFICS intervention can be considered as a compromise between delivering a standardized intervention program and modifications of the program to fit with the local needs in all participating countries [55].

The present paper aimed to describe the developmental process of the community-based IDEFICS intervention, providing valuable information for the development of obesity preventive intervention strategies in multi-centre settings.

## Conclusions

The IDEFICS project developed a community-based intervention program for the prevention of childhood obesity by following the intervention mapping heuristic. The intervention program is based on the socio-ecological approach and incorporated findings from formative research. The intervention targets both environmental and personal factors through the social contexts having an impact on young children. Findings from formative research provided the rationale for developing a general and standardized intervention framework. However, local and cultural adaptation was necessary to make the intervention feasible and to enhance deliverability in all participating countries, this way increasing the likelihood of program sustainability in the long term. The development of a multi-level and -behavioral intervention within a European context appeared to be a long process that needs to be done systematically. Sufficient time, human resources and finances need to be planned in advance to be able to develop an intervention that is evidence-based and culturally relevant.

## List of abbreviations used

SES: socio-economic status; IDEFICS: Identification and prevention of Dietary- and lifestyle-induced health EFfects In Children and infants; IMP: intervention mapping protocol; TV: television; IPM: intervention program manager

## Competing interests

The authors declare that they have no competing interests. The information in this document reflects the author's view and is provided as it is.

## Authors' contributions

All authors contributed to the development, implementation and evaluation of the IDEFICS intervention. All authors read and approved the final manuscript.
